# Adapting the nominal group technique for priority setting of evidence-practice gaps in implementation science

**DOI:** 10.1186/s12874-016-0210-7

**Published:** 2016-08-26

**Authors:** Nicole M. Rankin, Deborah McGregor, Phyllis N. Butow, Kate White, Jane L. Phillips, Jane M. Young, Sallie A. Pearson, Sarah York, Tim Shaw

**Affiliations:** 1 Sydney Catalyst Translational Cancer Research Cente, The University of Sydney, Level 6, 119-143 Missenden Road, Camperdown, NSW 2050 Australia; 2 Faculty of Health Sciences, The University of Sydney, Sydney, Australia; 3 Psycho-Oncology Co-operative Research Group, School of Psychology, The University of Sydney, Sydney, Australia; 4 Centre for Medical Psychology & Evidence-based Decision-making, The University of Sydney, Sydney, Australia; 5 Cancer Nursing Research Unit (CNRU), Sydney Nursing School, Sydney Local Health District and The University of Sydney, Sydney, Australia; 6 Faculty of Health, University of Technology Sydney, Sydney, Australia; 7 Sydney School of Public Health, The University of Sydney, Sydney, Australia; 8 RPA Institute of Academic Surgery, Sydney Local Health District, NSW Ministry of Health, Sydney, Australia; 9 Medicines Policy Research Unit, Centre for Big Data Research in Health, University of New South Wales, Sydney, Australia

**Keywords:** Health priorities, Implementation science, Methodology, Health services research, Lung neoplasms

## Abstract

**Background:**

There are a variety of methods for priority setting in health research but few studies have addressed how to prioritise the gaps that exist between research evidence and clinical practice. This study aimed to build a suite of robust, evidence based techniques and tools for use in implementation science projects. We applied the priority setting methodology in lung cancer care as an example.

**Methods:**

We reviewed existing techniques and tools for priority setting in health research and the criteria used to prioritise items. An expert interdisciplinary consensus group comprised of health service, cancer and nursing researchers iteratively reviewed and adapted the techniques and tools. We tested these on evidence-practice gaps identified for lung cancer. The tools were pilot tested and finalised. A brief process evaluation was conducted.

**Results:**

We based our priority setting on the Nominal Group Technique (NGT). The adapted tools included a matrix for individuals to privately rate priority gaps; the same matrix was used for group discussion and reaching consensus. An investment exercise was used to validate allocation of priorities across the gaps. We describe the NGT process, criteria and tool adaptations and process evaluation results.

**Conclusions:**

The modified NGT process, criteria and tools contribute to building a suite of methods that can be applied in prioritising evidence-practice gaps. These methods could be adapted for other health settings within the broader context of implementation science projects.

**Electronic supplementary material:**

The online version of this article (doi:10.1186/s12874-016-0210-7) contains supplementary material, which is available to authorized users.

## Background

Setting priorities in health services delivery and research is highly relevant in an era of scarce resources. In many settings, health researchers and clinicians participate in collaborative efforts to determine how best to implement evidence into routine clinical practice. Priority setting offers an important opportunity for collaborators to determine what evidence is relevant to implement, how best to do this, and where the greatest gains can be made in changing clinical practice [1]. There is a need to determine the most useful methods for eliciting priorities. Methods for priority setting in service delivery are already well developed [2, 3] but little attention has focused on how these methods could be usefully adapted for implementation science projects.

Few studies have addressed methods that specifically prioritise existing gaps between research evidence and clinical practice [1]. To date, only one cluster randomised controlled trial (RCT) in setting priorities for healthcare improvement in community settings has been published [4, 5]. The patient involvement intervention for chronic diseases was based on the Nominal Group Technique (NGT) and trialled in community workshops to set priorities with participants (professionals alone in the control group or professional and patients in the intervention group) [5]. The study found that the NGT intervention was effective and patient involvement significantly shifted priorities towards quality of care factors.

The NGT is an interpretive approach that engages stakeholders in interactive discussion to generate priorities [6, 7]. NGT has demonstrated validity, emphasizes considering all participants’ views equally and enables consensus on highly complex issues [8]. Consensus methods such as the NGT seek to overcome group or committee decision making that can be dominated by individuals or coalitions who have a vested interest in a specific outcome [9]. NGT is frequently used for problem identification; in helping to generate appropriate research questions; for development of solutions; and establishing priorities for action [8]. In cancer research, the NGT and modified Delphi techniques have been used to determine priorities in pancreatic cancer [10], haematological cancers [11, 12], colorectal cancer [13], colorectal cancer care coordination [14], psycho-oncology [15], cancer genomics [16] and with consumers in UK treatment centres [17]. To our knowledge, no studies have focused on methods for setting priorities about identified evidence-practice gaps in lung cancer care. Furthermore, there are few guides or resources specific to priority setting in ‘evidence to practice’ research [2, 18].

Our group had separately developed a set of seven evidence-practice gaps in lung cancer on the basis of a scoping review of the literature [19] and examining local data sources such as the Clinical Cancer Registry of New South Wales (NSW), Australia. Our primary aim was to test the relevance of these identified gaps with lung cancer health professionals using existing evidence-based approaches to priority setting (Additional file 1). Our second aim was to systematically develop a suite of robust, evidence based techniques, criteria and tools for use in implementation science projects. This paper describes the outcomes of the second aim. We show how the selected technique is relevant to implementation science, as most theoretical models and frameworks include pre-implementation engagement of stakeholders as a fundamental step in change processes [20–22]. The resulting tools and process were subsequently used in three focus groups with multidisciplinary teams (*n* = 42 participants) to fulfil our primary aim. Data outcomes from the focus groups are reported in a separate paper [23].

## Methods

### Selecting priority setting techniques

We conducted a literature search of journal articles and reports that specifically described priority-setting techniques in health research. We conducted three search strategies in Medline, PsychInfo and PubMed databases to:

- identify relevant health priority setting approaches or techniques that had been used in focus or small group settings;
- identify original research studies conducted in oncology settings, where oncology health professionals and/or consumers were the target audience for priority setting; and,
- locate relevant documents in the grey literature (i.e. reports and publications that may not be peer-reviewed) and web-based sources about group decision making tools used in health. This last search included checking the Cochrane Library (including the publication list of the *Cochrane Agenda and Priority Setting Methods Group*).

For all three search strategies, the inclusion criteria were those articles or sources published between January 2008 and December 2012 and published in English. The exclusion criteria were any studies that had a primary aim of conducting a comparative benefit or cost analyses of health services; we also excluded commentaries or letters to editors.

### Selecting criteria

The team conducted a literature search to identify and review existing criteria for rating evidence-practice gaps. We used the same databases and inclusion and exclusion criteria as described in the previous section. There are many and varied criteria used to assess priorities, which are generally categorised into three dimensions: public health benefit, feasibility and cost [18]. We focused on the first two dimensions and criteria options were collated from a range of sources [24, 25]. Cost criteria were not included as we had already excluded comparative benefit or cost analyses of health services in the previous section.

### Consensus approach to selecting relevant technique and criteria

A consensus process was undertaken to select: a) the most relevant technique; and, b) criteria to use in the rating tools. We formed an expert interdisciplinary consensus group comprising of cancer clinician-researchers and academic experts from multiple disciplines including nursing, psychology, health services research, epidemiology and clinical oncology. Results from the literature reviews were presented. To select the most relevant technique, the consensus group considered the relative advantages, disadvantages and/or the applicability of each one. They considered how optimal and practical each technique was in terms of equity of participation encouraged, time required to enact and the group size, stakeholder mix of disciplines and level of expertise required. To select the criteria for use in the rating tools, the consensus group focused on whether there was a clear definition for each criteria and how easily participants would be able to rate each gap against the criteria. The group discussed the criteria options until consensus was reached.

### Pilot testing of the process and tools

A pilot was conducted with key stakeholders (*n* = 7, including two medical oncologists and 5 health service researchers) to test the tools and the priority setting process. Minor adjustments were made to the content and formatting of the tools based on participant feedback. Participants indicated a maximum time of two hours should be allocated for the priority setting process. The pilot test confirmed that the priority setting technique and criteria were acceptable and feasible within this time-frame.

### Process and consensus evaluation

We asked the three lung cancer multidisciplinary teams (*n* = 42) to complete a brief 8-item survey to evaluate the process and provide feedback about whether consensus had been achieved.

## Results

The results are described in the following sections: selecting the technique; selecting the criteria; the resulting tools and the modified NGT process.

### Selecting the technique: outcomes of the literature search

**Fig. 1:**
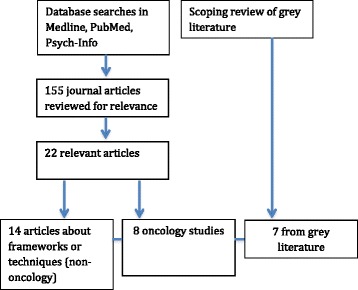
Literature review strategy for priority setting techniques

A total of 155 journal articles were identified from the first two search strategies; the abstract for each article was checked for relevance. Twenty-two articles relevant to the review were identified, which included 14 describing frameworks or techniques and eight that were original research articles in oncology. In the third strategy, five reports and two e-bulletins/newsletters from website sources were judged as relevant (see Fig. 1).

The following techniques were identified: the Nominal Group Technique (NGT) [2]; the Delphi technique [10]; the Six-Steps of Listening for Direction [18]; the Combined Matrix Approach (3D CAM) [26] and Dotmocracy [27]. Through consensus, it was agreed that the NGT would be most relevant to modify for the following reasons. It can engage clinicians and researchers in face-to-face structured priority setting groups. This approach allows information to be collected from health professionals working in ‘front line’ clinical areas [8]. NGT is time efficient and cost effective, as priorities can be elicited in a short time period (e.g. 2 h) and requires few resources, which appeals to busy clinicians and resource-limited research budgets [28]. The technique requires no preparation on the part of participants and yet their expertise is of fundamental importance in considering the issues for prioritization. Another advantage is that the results generated can be immediately shared with the group. This provides ample opportunities for further reflection and clarification before the priority setting group session concludes [28]. This is particularly relevant in settings where membership of clinical teams frequently changes.

### Selecting criteria: outcomes of the literature review and consensus

Using this same strategy described above, we identified seven journal articles and reports that listed relevant criteria options and three guides to consensus processes for selecting criteria [2, 18, 22]. Criteria options considered by the investigator team included those listed in: a) the Institute of Medicine (IOM) quality criteria (safety, effectiveness, patient-centered, timely, efficiency and equity) [29]; b) the 3D CAM criteria (disease burden, determinants of health, present level of knowledge, effectiveness and resource flows) [26], and c) the behavioural matrix in the Precede-Proceed model criteria (importance and changeable) [30, 31].

Through the consensus process, four criteria were selected against which the gaps could be rated. These were: 1) relevance to the local setting; 2) magnitude of the issue; 3) burden of suffering; and 4); amenable to change. Criteria 1-3 (relevance, magnitude and burden) were grouped under the heading of ‘significance’, so that results could later be collated and summarised more efficiently.

### The resulting tools

#### Tool 1: the priority setting matrix

This tool is used to elicit individual responses from participants, rating the gaps against the criteria using a matrix scoring sheet (Table 1). We adapted this tool from the 3D CAM [26]. The priority setting matrix places the evidence practice gaps in the left hand column and the criteria on the right. The matrix tool enables participants to privately rate each gap on a Likert scale ranging from 5 (most) to 1 (least) according to the four criteria. Likert scales have been used successfully in the past to rate priority items [32]. It should be noted that evidence practice gaps statements should be independent from each other and without multiple clauses.

**Table 1:** Evidence-practice gaps priority setting matrix

	Least			Most	
	1	2	3	4	5
	Criteria: significance			Criteria	
Evidence-practice gaps	Relevance to local setting	Magnitude of the gap (size)	Burden of suffering (severity)	Amenable to change	
1. Not all people with lung cancer receive timely diagnosis and referral for treatment					
2. People with potentially curable lung cancer who will benefit from active treatment do not always receive it					
3. People with advanced lung cancer who will benefit from palliative treatment do not always receive it					
4. People with lung cancer who are of an older age or with co-morbidities who may benefit from active treatment do not always receive treatment					
5. People with lung cancer who would benefit from review at a multidisciplinary team meeting are not always reviewed					
6. People with lung cancer have high levels of psychosocial needs which are not always being met					
7. Not all people with lung cancer who would benefit from early referral to palliative care services are offered this option					
8. Locally identified gap:					

Instructions for use: Please rate each evidence-practice gap in the left hand column according to the criteria in the right hand columns. Please rate each one according to a scale from 1 to 5

The same matrix tool can be used to elicit group responses for the purpose of discussing each gap and reaching consensus, and subsequently giving a rating to each one [23]. A ‘Dotmocracy’ approach is used to highlight common themes and reach consensus [27]. Gold and silver stars are allocated by each participant to reflect their highest priorities. Through facilitated discussion, the group is asked to discuss their individual ratings in a small group with each person nominating their top two (first ‘gold’ and second ‘silver’) gaps per criteria. This component is the small group trigger for discussion between participants of justifications for their ratings. Using the Dotmocracy approach can help small groups to determine if participant priorities align and to respond as appropriate until consensus is achieved.

#### Tool 2: validating priority selection through an investment exercise

Each participant receives $100 fake ‘dollars’ to spend across the gaps. This seeks to allow participants to make a global decision on resource allocation and also confirms whether the gaps are reflected in how participants would most like to invest in each gap. This acts as a cross check with the priorities selected in the matrix.

### The modified NGT process

Table 2 shows the original NGT steps and the modified NGT steps and a brief description for each.

**Table 2:** Comparison of original NGT steps and the modified process and brief descriptions

Original NGT		Modified NGT for priority setting	
Steps	Brief description	Steps	Brief description
Step 1: Generating ideas	Moderator directs participants to write their ideas in brief phases or statements	Step 1: Describe identified evidence practice gaps	Presentation about the evidence-practice gap literature review, with a brief summary for each gap provided
		Step 2: Present local data/information about the gaps	Presentation about national, jurisdictional and local data gathered to support the gaps
Step 2: Recording idea	Round robin feedback session to concisely record each idea	Step 3: Elicit feedback and record additional gaps identified by participants	Elicit feedback about relevance and appropriateness of evidence-practice gaps in the local service setting. Opportunity for participants to nominate additional local gaps
Step 3: Clarify, rank ideas	Participants express relative importance of each idea		
Step 4: Individuals vote privately to prioritise the ideas, using moderator-created criteria	Participants privately rate each gap	Step 4: Individuals vote privately to prioritise gaps, using moderator-created criteria	Participants privately rate each gap using Likert scale on the matrix tool
Step 5: Each participant selects the five most important items from the prioritised list	Each participant ranks top five ideas, with the highest receiving 5 and lowest 1	Step 5: Each participant selects the two most important gaps from the prioritised list	Each participant ranks top two gaps, with the highest receiving 2 and the lowest receiving 1
Step 6: Moderator creates tally sheet	The most highly rated ideas are the most favoured actions	Step 6: Focus group participants discuss ratings and moderator uses matrix tool as a tally sheet	In focus groups, participants share their ratings, speaking in turn to list their top two gaps and provide any clarification for their choices. Responses are recorded by a group facilitator on the matrix sheet in ‘Dotmocracy’ style
		Step 7: Whole group consensus	Small groups reform back into a larger group to review and discuss the gaps and resolve any differences to reach consensus
		Step 8. Investment exercise	Each participant asked to spend 100 fictitious dollars across each gap. Dollars are tallied and feedback provided to whole group

The modified NGT process developed for this project was subsequently used in focus groups and is described here as an exemplar. (Please note that the results of the focus groups are described in a separate paper [23]):

- Step 1: Describe identified evidence practice gaps
  In this step, an investigator team member (facilitator) gave a presentation about the approach used to review evidence about gaps in lung cancer care, read out a brief summary for each gaps and answered any participant questions.
- Step 2: Present local data/information about gaps
  This step included a presentation about national, jurisdictional and local data gathered to support the gaps. Where available, this was tailored to the local site to include analysis of local health district or hospital data collected in registries or administrative databases. This step was an adaptation of Lomas’ second step in the ‘listening model’ [6].
- Step 3: Elicit feedback and include additional gaps identified by stakeholders
  Participants were asked to provide feedback about the seven gaps and whether they were relevant in the local hospital setting and surrounding community. We provided an opportunity for participants to nominate any additional gaps and this was considered as elicitation and inclusion of ‘practice-based evidence’. Additional gaps were only included if consensus was reached within the group that the gap should be added.
- Step 4: Individuals vote privately to prioritise gaps, using moderator-created criteria
  The criteria were described by the facilitator and participants had an opportunity to seek any clarification before privately rating each gap. Participants were then asked to rate each gap for across the criteria listed above without sharing these with others. This was considered an important step as this private rating of items allowed participants to make their own considered judgements prior to reaching group consensus. This step also ensured that every member of the group had read and considered each gap in detail.
- Step 5: Each participant selects the two most important gaps from the prioritised list.
  At the conclusion of step 4, each participant was asked to select the two most important gaps from on the matrix tool for the two criteria of ‘significance’ and ‘amenable to change’, according to their highest scores. This step replicates the original NGT step 5 but is modified by selecting only two most important items instead of five.
- Step 6: Focus group participants discuss ratings and moderator uses matrix tool as a tally
  In this step, participants broke into small groups (maximum of seven participants in each) and shared their ratings for each gap. We pre-assigned participants into small groups that consisted of a mix of professional groups. Each group was asked to nominate a spokesperson to report back to the larger group and provide feedback about the choices made. To achieve equity within small groups, each person spoke in turn about their top two gaps and provided any clarification for their choices. All responses were recorded by a group facilitator on an A4/US letter-sized printed sheet of the matrix tool in ‘Dotmocracy’ style [27], assigning a gold and silver star to the highest priority gaps. Priorities were not weighted across the chosen top two gaps.
- Step 7: Whole group consensus
  At the conclusion of discussion, small groups reformed back into a larger group, compared ratings for differences and anomalies and resolved these to reach group consensus. The moderator led discussion about the gaps against the criteria of significance and amenability to change.
- Step 8. Investment exercise
  In the final step, participants were asked to spend $100 fake dollars across each gap, configured as five $20 notes. Eight A3/US Ledger-sized envelopes were placed on a table (one for each gap, including the eighth local gap) and participants were asked to spend their money in any way they might choose. Participants were able to configure the dollars in any way so long as all $100 was allocated (e.g. all $100 in one envelope or any configuration of $20 allocations across the envelopes). At the conclusion of the task, the investigator team tallied the dollars in each envelope and then reported back to the group about the proportions (total dollar values and per cent) spent on each gap.

### Participants’ feedback in the lung cancer exemplar

As a result of testing the methodology with the three lung cancer teams, we observed the following.

- Tools: The matrix tool was easy to complete and the Likert scale enabled participants to examine how they had comparatively rated each gap. By first completing the matrix tool privately, individual participants had an opportunity to focus on the evidence gaps and consider their responses according to the criteria. Using the same matrix for the group consensus process facilitated discussion in the small group setting. The investment tool of $100 fake dollars allocated across the gaps was a useful way to double check priorities after reaching consensus. The research team also observed that the investment exercise was a fun and engaging way to end the group session, with participants interested in the outcome and whether it was reflective of whole group consensus.
- Criteria: We found that the ‘amenable to change’ criteria was particularly meaningful for participants, as it allowed them to consider whether their current health service was capable of making change in the ‘gap’. Participation was a useful exercise for reflecting on current practice and what would be desirable to change in the future.
- Process evaluation: Thirty (of 42) (71 %) participants completed the evaluation survey. Results are shown in Table 3. There was strong agreement from participants that the priority setting process enabled full participation (93 %); was clearly communicated by the project team (86 %) and had adequate resources and time allocated (83 %). We asked participants if consensus of priorities had been reached via the process: 73 % responded ‘definitely yes’. Three participants provided a comment about the process: ‘fantastic brainstorming session; a very useful exercise; this was very novel and engaging’.

**Table 3:** Process and consensus evaluation for three focus group participants (*n* = 30)

	Definitely yes		Somewhat		Definitely no		Don’t know	
Process	*N*	%	*N*	%	*N*	%	*N*	%
Was the priority setting group scheduled and conducted in a way that enabled you to fully participate?	28	93	2	7	-	-	-	-
Did the project team clearly communicate information associated with the priority setting process?	25	83	5	17	-	-	-	-
Were adequate resources and time allocated to properly completing the priority setting process?	24	80	5	17	1	3	-	-
Would you be willing to participate in future priority setting activities?	22	73	7	23	-	-	1	3
Was this priority setting process beneficial in terms of identifying gaps in your local area?	21	70	8	27	-	-	1	3
Consensus					-	-		
Do you agree with the priorities that have been identified via this process?	22	73	7	23	-	-	1	3
In your opinion, are the priorities selected during this process representative of the broader views of cancer care stakeholders in your local area?	20	67	10	33	-	-	-	-
Will participation in this priority setting process lead to any changes within your organisation?	6	20	16	54	1	3	7	23

## Discussion

This study aimed to describe the methods for selecting and adapting a priority setting technique, selecting criteria and developing tools for implementation science projects. We sought to modify the NGT and relevant tools to allow the identified gaps to be rated according to agreed criteria. We believe this is the first study to demonstrate how to modify the NGT in setting priorities for evidence practice gaps within the context of implementation science projects. The selected technique, criteria, tools and process have drawn on evidence based approaches and we believe these will be highly relevant in other health care settings. A similar process was reported by Buckley and colleagues that assessed the effects of a research prioritization process to influence the research agenda in urinary incontinence [1]. Their team also noted that consensus methods for priority settings are still being developed and questions remain about whether this will impact on research priorities.

We consider that there is significant value in prioritising gaps in research evidence and where change should be targeted in clinical settings. Gaining stakeholder engagement is key in building effective relationships for adoption of intervention strategies [21]. The advantages of the NGT made it the most relevant choice for the context of this study and we noted many similar advantages to those reported by Harvey and Holmes in priority setting about pregnancy triaging in emergency departments [8]. We selected it over other techniques including a modified Delphi approach, which has been used in previous cancer research [10, 14, 16], as we sought to reach consensus about priority gaps in a workshop setting. The selected criteria enabled our team to seek participants’ views about what gaps were significant and amenable to change in their local setting, drawing on Green and Kreutzer’s behaviour matrix from the Precede-Proceed model [30].

It is worth reflecting on the modifications made to the NGT process. We modified the first two steps of NGT (generate and recording ideas) to become a presentation of an evidence-practice gaps summary, as the investigator team had already undertaken a significant amount of work to identify relevant gaps. By doing so, we sought to address the documented barrier of clinicians having insufficient time to read and assimilate research evidence [33]. Step 2 focused on presenting data at the jurisdictional (NSW) and local levels (local health district or hospital level) to support the selection of gaps. This adaptation draws on Lomas’ step of identifying and assembling data needed to help inform participants’ discussion [6] and we consider that this is an important component in appealing to a clinical audience whose priorities are likely to focus on the technical aspects of disease management [4] and epidemiological data. Step 3 provided participants with an opportunity to discuss the gaps and elicit any additional gaps for the local setting. This step allowed for group discussion and generation of new gaps of relevance. We replicated Steps 4 and 5 of the original NGT model to be a private ranking test of items, however, in Step 5 we reduced the number of top gaps from five to two. This reduction was due to time constraints, and with having eight gaps to assess, we considered this a reasonable adjustment to make to the process. An alternative is to maintain original NGT step to rank the 5 top items or to rank items on a Likert scale.

Our modifications include additional opportunities for group discussion (Steps 6 and 7). In a subsequent study to test success factors about coordinated cancer in care [34], we found in Step 6 that rating items using the top three priorities as scores (3 = top rated item, 2 = second highest item, 1 = third item) was an easier method for reporting results where there were multiple break out groups and reaching whole group consensus. The addition of the investment exercise in Step 8 was helpful way of reflecting on the group’s priorities. In the event that this step reveals different priorities, we recommend considering a return to the group consensus process (Step 7) to determine with the large group how best to resolve divergent responses. Facilitators who are faced with a lack of consensus amongst participants should focus on the provision of an ‘equal voice’ for all participants, rather than expecting a specific outcome. While reaching consensus may be a lengthy process and contentious, there is limited evidence to suggest that gaining stakeholder buy-in results in increased likelihood of success. This is preferable in health settings to ‘majority rules’ where dominant views overrule the minority, creating ‘winners and losers’ thus potential disenfranchising participants and leading to poorer outcomes.

### Study limitations and future directions

The limitations of this study are that we modified the NGT process and tools with a specific purpose of testing evidence-practice gaps in lung cancer with multidisciplinary teams of clinicians working across three hospital settings. Whilst we have used a robust and evidence-based approach to reviewing existing priority setting techniques, it is by no means the only option. Researchers will seek to adapt techniques, criteria, tools and processes to suit their own research aims and local context. However, given the paucity of tools available to researchers working in implementation science [35], we consider that the methods described in this paper make a significant enhancement to the knowledge base. Since conducting this study, we have been approached by three research teams to use this methodology in different health settings. We have utilised this methodology in a project to define success factors for coordinated care in cancer [34] on behalf of the Cancer Institute NSW. Future directions include opportunities to replicate the process with consumers, or with combined groups of health professionals and consumers, similar to the cluster RCT conducted by Boivin and colleagues [4].

## Conclusions

In conclusion, this study shows that existing priority setting techniques and tools can be successfully adapted for use in implementation science projects. The modified NGT process and tools can be used by collaborations of researchers and clinicians who seek to identify priorities for research within a particular health condition. The modified NGT approach is practical, user-friendly and easy to conduct in small groups of clinicians. The study provides a methodology for how to prioritize evidence-practice gaps in other health settings, within the broader context of implementation science projects.

## Additional file

Additional file 1: An exemplar of evidence-practice gaps in lung cancer. (DOCX 14 kb)

## Abbreviations

RCT: Randomised controlled trial
NGT: Nominal group technique
NSW: New South Wales
3D CAM: Combined matrix approach
IOM: Institute of medicine
