# Supplementary material for: Adapting the nominal group technique for priority setting of evidence-practice gaps in implementation science
Source: BMC Med Res Methodol. 2016 Aug 26;16(1):110. doi: 10.1186/s12874-016-0210-7 (PMC5002198; doi:10.1186/s12874-016-0210-7)
Supplement: Additional file 1: — An exemplar of evidence-practice gaps in lung cancer. (DOCX 14 kb) [file 12874_2016_210_MOESM1_ESM.docx]

**Supplementary file 1: An exemplar of evidence-practice gaps in lung cancer**

Sydney Catalyst Translational Cancer Research Centre was established in July 2011 as a multidisciplinary and multi-institutional virtual consortium of researchers and clinicians from more than 20 member organisations (hospitals, research centres and The University of Sydney). These organisations are spread across metropolitan Sydney and regional New South Wales, Australia. A flagship ‘evidence into practice’ program in lung cancer commenced in July 2012 and adapted the Knowledge to Action Cycle as its theoretical foundation [21]. We conducted a literature synthesis of the evidence-practice gaps in lung cancer as the first program component. The investigator team simultaneously commenced with activities to engage with clinical stakeholders from the member organisations. We developed a partnership with lung cancer clinicians located in three hospitals (two urban, one regional), with the intention of conducting implementation research projects that would target the evidence-practice gaps and seek to close the gaps. Thus, the second flagship program component focused on priority setting with each team. We worked with the clinical leads to identify health professional participants within the hospital setting and the community. This included pathology, radiology, primary and community care, respiratory medicine, cardio-thoracic surgery, clinical oncology, nursing, allied health, psycho-oncology, data and clinical trials managers. We purposively sought to include both health professionals who had direct contact with patients (including surgical ward nurses and care coordinators) and those who did not (for example, pathologists and data managers). We report on the data outcomes in a separate publication [23].
